# Selection of Transcripts Affecting Initial Growth Rate of Rice Backcrossed Inbred Lines Using RNA Sequencing Data

**DOI:** 10.3389/fpls.2018.01880

**Published:** 2018-12-20

**Authors:** Akari Fukuda, Tatsuro Hirose, Naohiro Aoki, Satoshi Kondo, Madoka Yonekura, Tomomori Kataoka, Chikara Ohto, Atsushi J. Nagano

**Affiliations:** ^1^Central Region Agricultural Research Center, National Agriculture and Food Research Organization, Joetsu, Japan; ^2^Graduate School of Agricultural and Life Sciences, The University of Tokyo, Tokyo, Japan; ^3^Agriculture and Biotechnology Business Division, Toyota Motor Corporation, Miyoshi, Japan; ^4^Kyushu Okinawa Agricultural Research Center, National Agriculture and Food Research Organization, Fukuoka, Japan; ^5^T-Frontier Division, Toyota Motor Corporation, Toyota, Japan; ^6^Faculty of Agriculture, Ryukoku University, Otsu, Japan

**Keywords:** eQTL, LASSO, *Oryza sativa*, qLTG3-1, QTL, RNA-Seq, *SG1*, transcriptome

## Abstract

Seedling growth is an important factor for direct seeding of rice. However, the genetic and transcriptomic factors involved in this process are largely unknown. In this study, transcripts affecting shoot weight were identified in rice (*Oryza sativa* L.) using RNA sequencing (RNA-Seq) data from 20 backcrossed inbred lines (BILs) and their parental cultivars. The selection frequency of the genes for the regression model was determined using repeated analysis of random subsets of the transcriptome. The *qLTG3-1*gene, controlling low-temperature germinability, and short grain 1 gene (*SG1*), known to decrease organ elongation, showed high frequency. The quantitative trait loci (QTLs) analysis performed for BILs revealed that *qLTG3-1* was included in the QTLs for shoot weight but *SG1* was not. No nucleotide polymorphisms were found in the coding region of *SG1* in either of the parental cultivars. Quantitative real-time PCR showed that *SG1* expression was negatively correlated with shoot weight for all 104 BILs analyzed in this study. Expression QTL (eQTLs) analysis showed an eQTL for *SG1* expression located in the same region as the QTL for shoot weight. However, no eQTLs were detected on the same chromosome as *SG1*, suggesting that nucleotide polymorphisms around the gene do not affect its expression in analyzed growth stage. Overall, these results indicate that RNA-Seq is a useful tool for identifying transcripts that can be related to seedling growth rate.

## Introduction

Direct seeding of rice (*Oryza sativa* L.) is less laborious and costly than transplanting but has some limitations. Early seedling growth, for example, is an essential process during direct seeding, and delayed growth results in increased seedling mortality (Jones and Peterson, 1976; Ogiwara and Terashima, 2001). Therefore, although seedling growth rate and other agronomical traits have been suggested to be regulated by complex genetic and environment interactions, it is important to elucidate the genetic factors involved in enhancing these traits, as this will allow selecting preferable traits during rice breeding to improve the efficacy of direct seeding. One tool used to identify genetic factors affecting agronomical traits is quantitative trait loci (QTLs) analysis, which was previously performed to evaluate traits affecting seedling vigor (Iwata et al., 2010; Abe et al., 2012; Yano et al., 2012; Fukuda et al., 2014). However, QTLs analysis is laborious, time consuming, and only reveals genomic mutations; in some cases, it also involves significant genetic bias.

Another method to elucidate the physiological traits involved in a process or phenotype is transcriptomic analysis based on RNA sequencing (RNA-Seq), which measures gene expression in an unbiased genome-wide manner. This technique has been used to reveal genes related to many endogenous and environmentally influenced agronomical traits. This includes analysis of differential gene expression in groups of plants subject to specific environmental conditions (Nagano et al., 2012; Plessis et al., 2015), which has been used to establish statistical models to predict gene expression dynamics under various field conditions (Nagano et al., 2012; Matsuzaki et al., 2015). Transcriptomics has also been used to predict individual plant traits. In maize (*Zea mays* L.), “expression biomarkers” calculated using a linear regression model of transcriptomic gene expression can be used to predict nitrogen sensitivity status (Yang et al., 2011). However, this kind of transcriptomic analysis has not yet been applied to study seedling growth rate in rice.

In the present study, rice cultivars with varying shoot weights were subject to RNA-Seq to identify genes whose expression can influence seedling growth rate. Backcrossed inbred lines (BILs) were produced with Arroz da Terra, which has a rapid initial growth rate, as the donor parent and Ouu365 as the recipient parent (Fukuda et al., 2014). Both parental cultivars and BILs were analyzed and, interestingly, all analyzed plants had different shoot weights. To our knowledge, this is the first time that an unbiased, statistical approach has been used to select genes which expression is related to shoot weight/seedling growth in rice.

## Materials and Methods

### Plant Materials and Growth Conditions

The parental strains Ouu365 and Arroz da Terra, as well as the BILs used in the present study, were developed as described previously (Fukuda et al., 2014). Seeds were sterilized for 1 h in 50-fold diluted antiformin, rinsed three times in tap water, and imbibed in water at 30°C for 2 days in an incubator. Twenty-four germinated seeds per line were sown on floating plates (Fukuda et al., 2012), which were floated on hydroponic culture solution containing 0.35 mM NH_4_NO_3_, 0.18 mM Na_2_HPO_4_, 0.27 mM K_2_SO_4_, 0.46 mM MgSO_4_, 0.36 mM CaCl_2_, and 0.05 mM FeSO_4_ (Hayashi and Chino, 1986). The pH of the hydroponic culture solution was adjusted to 5.5, and the solution was replaced every 2 days. The seedlings were grown in a chamber LH-240 (Nippon Medical and Chemical Instruments, Osaka, Japan) for 14 days at 20°C under a 12-h artificial light and 12-h dark cycle. The light was radiated with 64–79 μmol m-2 s-1 of photosynthetically active radiation by the neutral white fluorescent lamp. The positions of the floating plates were changed every 2 days in the chamber.

### QTL Analysis

The parental cultivars and all 104 BILs were grown in the chamber as described above. For all lines, growth experiments were repeated three times in independent periods. The seedlings of 104 BILs and parental cultivars were harvested 14 days after germination and oven dried at 80°C for 2 days. Seeds and roots were then removed from each seedling, and shoot dry weight per line was determined. The mean value of three biological replicates of shoot dry weight was used for QTL analysis. The genotypes of BILs were previously analyzed using 124 simple sequence repeat markers (Fukuda et al., 2014). Information on the simple sequence repeat markers is listed in GRAMENE.^1^ We also used a marker which detect 71-bp deletion in the *qLTG3-1* gene (RAP ID: Os03g0103300) on chromosome 3 with the S103a primers (Fujino et al., 2008). While the Ouu365 allele presented the 71-bp deletion in its open reading frame (ORF), Arroz da Terra did not (Fukuda et al., 2014). Additionally, we designed a PCR restriction fragment length polymorphism marker (PCR-RFLP marker) that detect a single nucleotide change in the –1948 bp region upstream the translational start site of *SG 1* gene (RAP ID: Os09g0459200) on chromosome 9. The PCR fragment produced using a specific primer set (5′-GGGACGTGATAACCGACTCA-3′ and 5′-TTCAGGTCACCTAGCCCATC-3′) was digested with the restriction enzyme *Hae*III (Takara Bio). In this analysis, the Arroz da Terra allele would be digested, while that of Ouu365 would not thereby allowing their discrimination. Linkage analysis was performed using MAPMAKER/EXP 3.0 (Lander et al., 1987). The frequencies of recombinations between two markers were converted to genetic distance using the function of Kosambi (1943). The QTLs were determined using the software QTL Cartographer 2.5 (Wang et al., 2010).^2^ Composite interval mapping was performed to determine QTLs with forward backward regression. A threshold LOD score of each trait was determined at a significance level of 0.05, using 1000 permutation tests.

**FIGURE 1:**
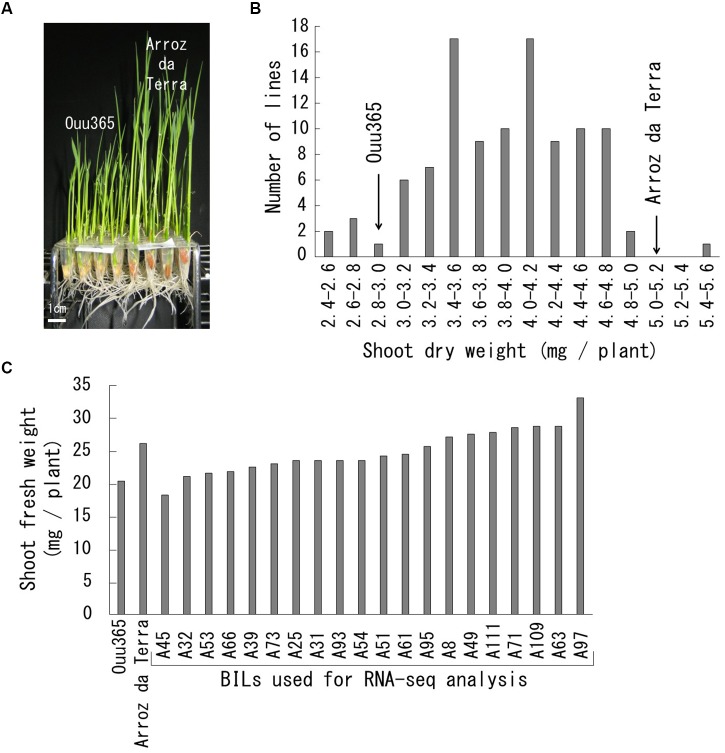
Growth rate of Ouu365, Arroz da Terra, and BILs. **(A)** Ouu365 and Arroz da Terra seedlings 14 days after germination. **(B)** Frequency distribution of the shoot dry weights of 104 BILs. Arrowheads indicate the mean values of three biological replicates in Ouu365 and Arroz da Terra. **(C)** Shoot fresh weight of Ouu365, Arroz da Terra, and BILs used for RNA sequencing analysis.

**FIGURE 2:**
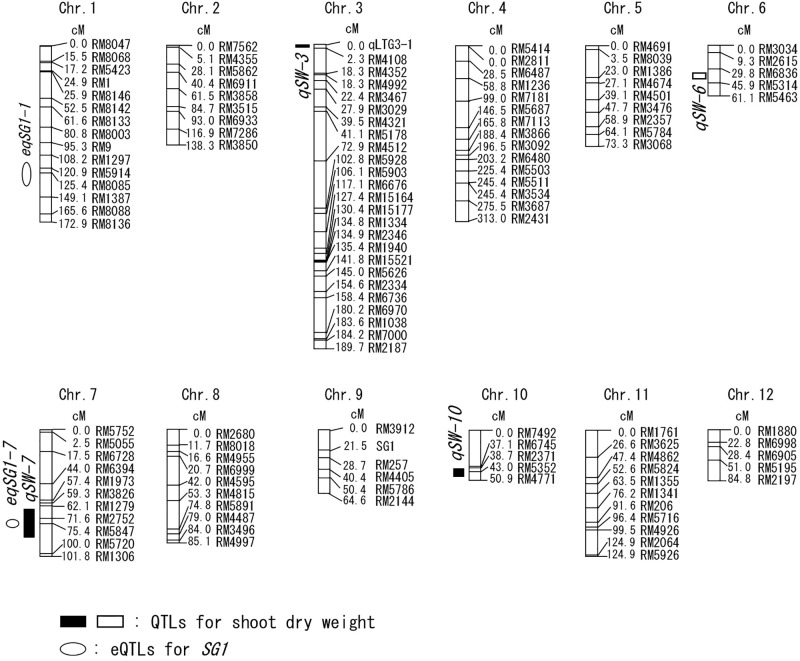
Chromosomal locations of the QTLs for shoot dry weight and the expression QTLs (eQTLs) for short grain 1 (*SG1*) gene. The black or white boxes indicate the positions of the QTLs for shoot dry weight. The white circles indicate the positions of the eQTLs for *SG1*. The Ouu365 cultivar has the positive allele for the QTLs indicated by white symbols. The Arroz da Terra cultivar has the positive alleles for the QTLs indicated by black symbols. The names of markers and the genetic map position (cM) are represented on the right of the corresponding chromosome. The positions of *qLTG3-1* and *SG1* are indicated on chromosome 3 and 9, respectively.

### RNA Isolation and RNA-Seq

The parental cultivars, Ouu365 and Arroz da Terra, and 20 BILs with variable initial growth rates were selected for RNA-Seq analysis. Twenty-four seedlings per line were grown as described above. The seeds and roots of the seedlings were removed 14 days after germination. After weighing, fresh shoots were frozen in liquid nitrogen and stored at −80°C until analysis. Total RNA was extracted from 24 shoot samples in each line using an RNeasy mini Kit (Qiagen, Hilden, Germany) according to the manufacturer’s instructions. The quality and quantity of RNA were assessed with a 2100-Bioanalyzer (Agilent Technologies, Santa Clara, CA, United States). One biological replicate was conducted in each line. The 22 RNA samples were used to produce sequencing libraries with the TruSeq RNA LT Sample Preparation Kit v2 (Illumina Inc., San Diego, CA, United States), which were sequenced as 100-bp single-end reads in the Illumina HiSeq 2000 platform. The resulting Fastq files have been deposited into the DNA Data Bank of Japan Sequence Read Archive (DRA; accession no. DRA006312). Sequence data were mapped to the *Oryza sativa*-Nipponbare-Reference-IRGSP-1.0 genome assembly (Oryza sativa.IRGSP-1.0.24.dna.toplevel.fa.gz^3^) and gene set (Oryza sativa.IRGSP-1.0.24.gtf.gz^4^) using TopHat2 (Trapnell et al., 2009; Kim et al., 2013). Transcript expression was calculated as fragments per kilobase per million (FPKM) based on the number of uniquely mapped reads that overlapped exon regions. Sequencing of RNA and estimation of transcript expression were performed by Takara Bio Inc. (Kusatsu, Shiga, Japan).

### Gene Selection Frequency

**FIGURE 3:**
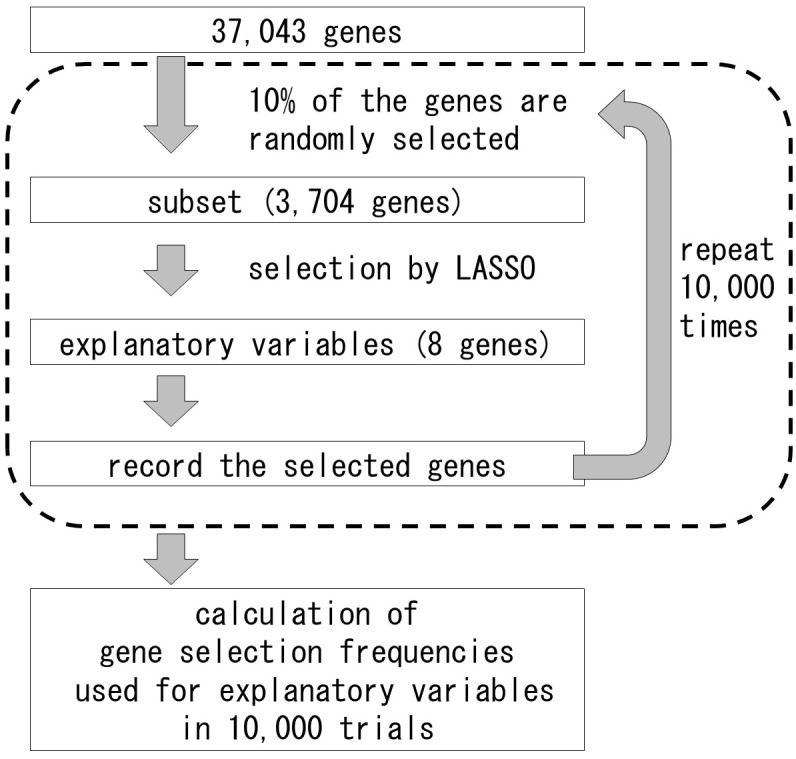
Method to assess gene selection frequencies for the explanatory variables using LASSO.

In general, the differential expression analysis between the opposite extreme trait groups need replicates to decrease the noise. However, most traits of organisms are continuous and could not be separated into two opposite groups. Furthermore, it is difficult to take replications in the natural situations, because of the samples under certain conditions could be collected only once. Therefore, we tried the new statistical approach to select the transcripts affecting the gradient traits. In this study, one sample represented one state, and it was used for statistical analysis without replicate. To determine which transcripts were correlated with shoot fresh weight and the selection frequencies for the explanatory variables, we used the 37,043 genes whose average expression was above 0.01 in our RNA-Seq analysis data. Overview of the method to assess the selection frequencies of the genes for the explanatory variables is presented on Figure 3. Gene expression values were log2-transformed after adding 0.01 to each FPKM value. Explanatory variables were determined using a L1-regularized linear regression model constructed with LASSO (Tibshirani, 1996). To assess gene selection frequencies, we repeatedly evaluated explanatory variables using subsets of the transcriptome. Among the 3,704 genes (10% of the transcriptome) randomly selected and used as input variables for LASSO, eight presented non-zero coefficients in LASSO (Tibshirani, 1996), and were, therefore, designated as explanatory variables. The subset selection and explanatory variables determination steps were repeated 10,000 times. Gene selection frequency was defined as the ratio of trials in which the gene could be used as an explanatory variable to the total number of trials. The analysis was conducted with the R glmnet package (R Core Team, 2015).^5^ A list of selected genes with frequencies larger than 0.01 is provided in Supplementary Table 1. Descriptions of high frequency genes were obtained from The Rice Annotation Project Database (RAP-DB)^6^ and EnsemblPlants.^7^

### Sequencing of Short Grain 1 Gene (*SG1*)

The genomic sequences of Ouu365 and Arroz da Terra, including the –2108 bp upstream region and ORF of *SG1*, were amplified by PCR with primers 5′-GGGACGTGATAACCGACTCA-3′ and 5′-CCCCACTGTACGTTCTCTCC-3′. Amplicons were isolated with an Illustra ExoProStar kit (GE Healthcare, Uppsala, Sweden), according to the manufacturer’s instructions, and sent to Fasmac Co. (Kanagawa, Japan) for sequencing.

### Quantitative Real-Time PCR (qRT-PCR)

For the qRT-PCR, the seedlings of 104 BILs and parental cultivars were grown in the chamber as described in Plant materials and growth conditions. All lines were grown three times in independent periods. Total RNA was extracted from shoot samples as described above, and used (1 μg) to synthesize cDNA with the PrimeScript RT reagent Kit with gDNA Eraser (Takara Bio) according to the manufacturer’s instructions. The resulting cDNA was used for PCR amplification in a Thermal Cycler Dice Real Time System III with SYBR Premix Ex Taq II (Takara Bio). The cDNA for *SG1* (RAP ID: Os09t0459200-01) was detected with the OA045647 primer set (Takara Bio). Three biological replicates and three technical replicates were analyzed for each sample. To determine *SG1* mRNA copy numbers, *SG1* standard fragments were amplified using Ouu365 cDNA as template and primers 5′-CGACCAGCTGATCTCCAAG-3′ and 5′-CATTTTTACTGGCCCTTCCA-3′. The standard fragment content was quantified with a Qubit fluorometer (Thermo Fisher Scientific, Waltham, MA, United States). Copy numbers of the standard fragments were determined by their concentration and molecular weight. Standard curves were constructed using a dilution series of the fragments solutions. The results obtained for each of the cDNAs were log2 transformed, and the mean value of the three biological replicates was used for the analysis of QTLs as described above.

### Statistical Analyses

Significant differences in shoot weights and expression values among genotypes were evaluated by analysis of variance (ANOVA) in R software, considering *P* < 0.05 as significant. Pearson’s product-moment correlations between expression values and shoot weights were also calculated in R software, considering *P* < 0.05 as significant.

## Results

### Shoot Growth Rates and QTLs in BILs

Shoot growth rates of Ouu365, Arroz da Terra, and of the 104 BILs (Fukuda et al., 2014) were measured 14 days after germination (Figure 1). The mean value of shoot dry weight was significantly larger in Arroz da Terra (5.11 mg) than in Ouu365 (2.91 mg) (Figure 1A; *P* = 0.0056), while that of the 104 BILs ranged from 2.52 to 5.47 mg (Figure 1B). The 104 BILs presented four QTLs (*qSW-3*, *qSW-6, qSW-7*, and *qSW-10*) for shoot dry weight on chromosomes 3, 6, 7, and 10, respectively (Table 1 and Figure 2). Three QTLs *qSW-3*, *qSW-7*, and *qSW-10*, correlated with increased shoot dry weight were from the Arroz da Terra parent, while *qSW6* increased shoot weight through the Ouu365 allele.

**Table 1:** QTLs for shoot dry weight per plant detected in the BILs.

Trait	QTL	Chr.	Nearest marker	LOD	Additive effect	*R*^2^ (%)
Shoot dry weight	*qSW-3*	3	qLTG3-1	3.73	0.21	10.1
	*qSW-6*	6	RM6836	3.35	−0.28	14.1
	*qSW-7*	7	RM5847	4.03	0.21	11.1
	*qSW-10*	10	RM5352	3.36	0.20	9.6

*The chromosome (Chr.) on which the QTL is located is provided. The additive effect is the effect of substituting an Arroz da Terra allele for an Ouu365 allele. A positive value indicates that Arroz da Terra has the allele that increases trait value. R^2^ indicates the variation explained by each putative QTL.*

### RNA-Seq Analysis and Gene Selection Frequency

To identify the transcripts associated with the initial growth rate, the parental cultivars and 20 BILs with varying shoot weights were analyzed by RNA-Seq (Figure 1C). An average of 41.6 million reads per sample was obtained, and about 40.0 million reads per sample, corresponding to 96.1% of the total reads, were successfully mapped to the Os-Nipponbare-Reference-IRGSP-1.0 genome. Our analysis to determine the gene selection frequencies for the explanatory variables of shoot weight identified 158 genes with frequencies larger than 0.01 (Supplementary Table 1). The expression of these 158 genes was significantly correlated with shoot fresh weight (*P* < 0.05).

### High Frequency Genes Located at the QTLs for Shoot Weight

**FIGURE 4:**
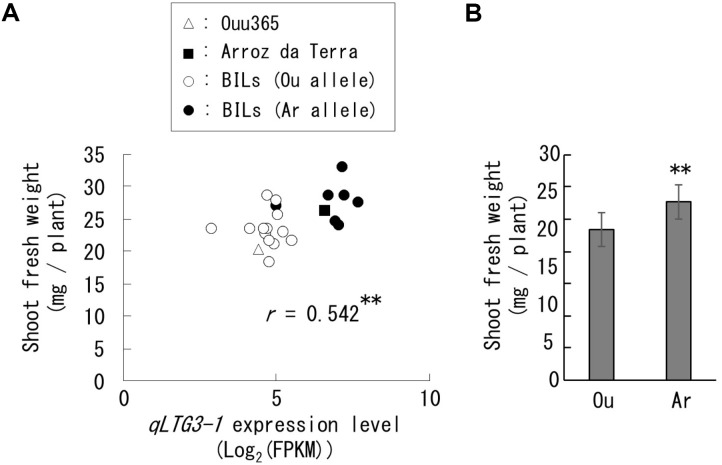
Expression of *qLTG3-1* and shoot fresh weight in the lines used for RNA sequencing. Each symbol indicates the parental cultivars and BILs that have the Ouu365 (Ou) or Arroz da Terra (Ar) allele for *qLTG3-1.* **(A)** Relationships between *qLTG3-1* expression and shoot fresh weight. The *r*-value indicates the correlation coefficient. ^∗∗^*P* = 0.0092 (*n* = 22). **(B)** Mean value of the shoot fresh weight in each allele for *qLTG3-1* with standard deviations. ^∗∗^*P* = 0.0026 (Ou: *n* = 14, Ar: *n* = 8).

Only two, three, six, and zero of the 158 high-frequency genes identified in our RNA-Seq analysis were present in the QTLs *qSW-3, qSW-6, qSW-7*, and *qSW-10*, respectively (Supplementary Table 1). Among these, only one *qSW-3* gene and five *qSW-7* genes were differentially expressed between Ouu365 and Arroz da Terra alleles (Supplementary Figure 1). The low-temperature germinability locus *qLTG3-1* (RAP ID: Os03g0103300) located at *qSW-3* is of particular interest, as it is known to accelerate germination speed (Fujino et al., 2008). The functional *qLTG3-1* was found in Arroz da Terra, while Ouu365 had a deletion in the ORF of *qLTG3-1* (Fukuda et al., 2014). In the lines used for RNA-Seq, *qLTG3-1* expression was significantly and positively correlated with shoot fresh weight in the lines used for RNA-Seq (Figure 4A). Furthermore, *qLTG3-1* expression and shoot fresh weights of the lines containing the Arroz da Terra allele were significantly higher than those of the lines containing the Ouu365 allele (*P* = 7.8e-07 and *P* = 0.0026, respectively) (Supplementary Figure 1A and Figure 4B).

### Analysis of a Functional Gene Not Located at the QTLs for Shoot Weight

**FIGURE 5:**
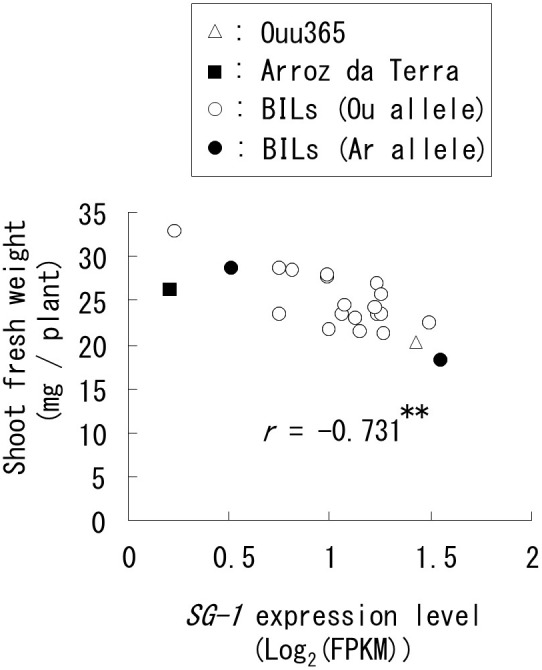
Relationships between short grain 1 (*SG1*) expression and shoot fresh weight in the lines used for RNA sequencing. Each symbol indicates the parental cultivars and BILs that have the Ouu365 (Ou) or Arroz da Terra (Ar) allele in the upstream region (−1948 bp) from the translational start site of *SG1*. The *r*-value indicates the correlation coefficient; ^∗∗^*P* = 0.00011 (*n* = 22).

In the list of high frequency genes that were not located at the QTLs for shoot weight, we recognized a functional gene known to decrease organ elongation, i.e., *SG1* (RAP ID: Os09g0459200; Nakagawa et al., 2012). In our analysis, *SG1* expression was significantly and negatively correlated with shoot fresh weight in the lines used for RNA-Seq (Figure 5). The genomic sequence of *SG1* in Ouu365 and Arroz da Terra were also compared to identify any nucleotide substitutions or insertion/deletion in the coding region; however, none were detected. However, two substitutions (−1948 A to G and −2038 T to C) were detected in Arroz da Terra in the region upstream from the translational start site. Interestingly, these substitutions did not appear to significantly alter *SG1* expression in the lines used for RNA-Seq (*P* = 0.17).

### *SG1* qRT-PCR in the 104 BILs

**FIGURE 6:**
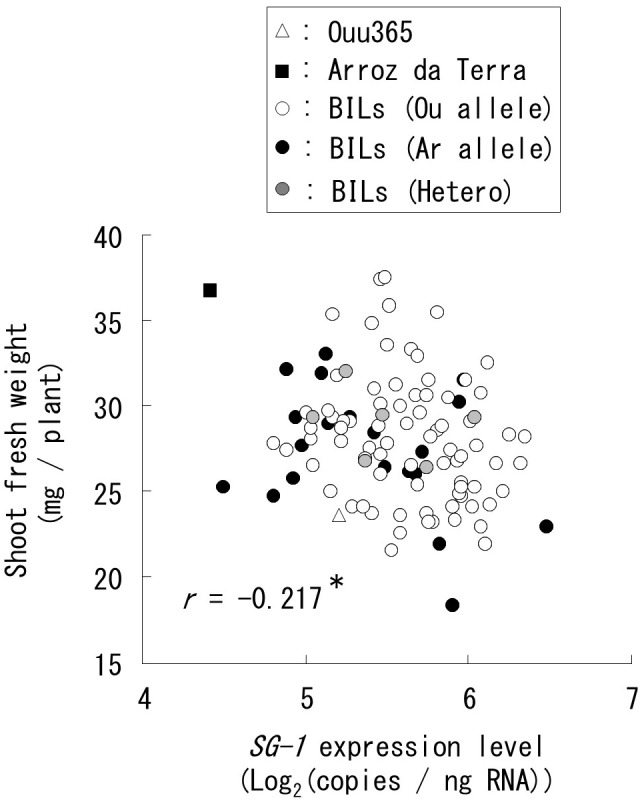
Relationships between short grain 1 (*SG1*) expression and shoot fresh weight for all the 104 BILs were analyzed by quantitative real-time PCR. Each symbol indicates the mean value of *SG1* expression levels and shoot fresh weights in three biological replicates. The shapes and colors of symbols indicate the parental cultivars and BILs that have the Ouu365 (Ou), Arroz da Terra (Ar), or heterozygous (Hetero) alleles in the upstream region (−1948 bp) from the translational start site of *SG1*. The *r*-value indicates the correlation coefficient; ^∗^*P* = 0.027 (*n* = 104).

To further evaluate the correlation between *SG1* expression and shoot weight, we analyzed all 104 BILs, including those that were not used for RNA-Seq. Our results showed that *SG1* expression was significantly and negatively correlated with shoot fresh weight (Figure 6). However, the variation in *SG1* expression was not explained by the genotype of the upstream region in the 104 BILs (*P* = 0.075). Thus, to identify the chromosomal region that influences *SG1* expression, an expression QTL (eQTL) analysis was conducted. Our results distinguished two eQTLs (*eqSG1-1* and *eqSG1-7*) located on chromosomes 1 and 7 that are correlated with decreased *SG1* expression by the Arroz da Terra allele (Table 2 and Figure 2). Notably, *eqSG1-7* is located in the same chromosomal region as the *qSW-7* QTL for shoot weight. No eQTL was detected on the same chromosomal regions as the *SG1* gene.

## Discussion

Evaluating the connections between genetic or transcriptomic changes and phenotypic manifestation is essential for understanding the biology of an organism. Techniques such as QTL and RNA-Seq analyses provide the means to evaluate the genetic code and expression profile reflecting specific morphological and physiological traits. Although QTL analysis was previously performed to evaluate traits affecting rice seedling size and growth rate (Iwata et al., 2010; Abe et al., 2012; Yano et al., 2012; Fukuda et al., 2014), RNA-Seq has never been applied to study the rice transcriptome for initial growth rate. Thus, we analyzed 20 BILs and their parental cultivars using RNA-Seq to identify genes that influence initial growth rate.

One of the main findings of this study was that *SG1* appears to be functionally significant during initial growth rate specification. It has been shown that *SG1* upregulation decreases organ elongation (Nakagawa et al., 2012), although it was not clear if *SG1* expression differed among natural rice lines. Our results indicate that *SG1* expression is negatively correlated with shoot weight in the lines used for the RNA-Seq. Moreover, our qRT-PCR analysis showed that this negative correlation was not only present in the lines used for RNA-Seq, but in all 104 BILs, suggesting that *SG1* expression levels might affect the growth rate of rice seedlings. These results also highlight an important technical finding as it appears that RNA-Seq analysis of 22 random and representative rice lines were useful to predict functional genes for the initial growth rate of a much larger population.

**Table 2:** Expression QTLs (eQTLs) for the short grain 1 (*SG1)* gene detected in the BILs.

Trait	QTL	Chr.	Nearest marker	LOD	Additive effect	*R*^2^ (%)
*SG1* expression	*eqSG1-1*	1	RM5914	3.74	−0.17	12.4
	*eqSG1-7*	7	RM5847	3.02	−0.13	8.6

*The chromosome (Chr.) on which the eQTL is located is provided. The additive effect is the effect of substituting an Arroz da Terra allele for an Ouu365 allele. A positive value indicates that Arroz da Terra has the allele that increases trait value. R^2^ indicates the variation explained by each putative eQTL.*

Notably, *SG1* overexpression was previously reported to induce a dwarf phenotype and to decrease the response to brassinosteroid (Nakagawa et al., 2012). However, natural *SG1* mutations have not been reported, and no nucleotide polymorphisms were found in the coding region of *SG1* in the parental cultivars Ouu365 and Arroz da Terra in the present study. Although the upstream promoter region of *SG1* contained nucleotide substitutions, these changes did not affect *SG1* expression and no eQTLs were located on the same chromosomal region as the *SG1* gene. These results indicate that the differences in *SG1* expression level between the Ouu365 and Arroz da Terra lines are not greatly affected by genomic substitutions upstream of the gene. Furthermore, this analysis showed that RNA-Seq analysis effectively detects transcripts that do not have genomic mutations. It is important to note, however, that an eQTL for *SG1* and one of the known QTLs for shoot weight were co-located in a region of chromosome 7, suggesting that this area might contain transcription factors that regulate *SG1* expression. Additional work is necessary to identify the regulatory factor(s) responsible for the observed differences in *SG1* expression.

Another important aspect of our study involved comparing the QTLs for BILs’ shoot weight and the high frequency selected gene *qLTG3-1*, which affected germination speed, and is included in the QTL region of chromosome 3. Arroz da Terra is reported to have the functional *qLTG3-1* allele, while the Ouu365 allele contains a 71-bp loss of function deletion in its ORF (Fujino and Iwata, 2011; Fukuda et al., 2014). The functional *qLTG3-1* allele is known to accelerate germination speed (Fujino et al., 2008) and pre-harvest sprouting (Hori et al., 2010), although the effects of *qLTG3-1* after the germination stage are unclear. In the present study, *qLTG3-1* alleles resulted in differences in gene expression and shoot weight, suggesting that *qLTG3-1* affects the seedling growth rate after the germination stage. Thus, combined analysis of QTLs and transcriptomic sequencing effectively detects QTL candidate genes, as previously reported (Yano et al., 2012; Chen et al., 2016). Indeed, QTLs and microarray analyses of substitution lines carrying a QTL allele were previously used to detect candidate genes encoding gibberellin oxidases that are involved in rice shoot length (Yano et al., 2012). Moreover, RNA-Seq of bulk recombinant inbred lines that had extremely high or low grain chalkiness was also used to evaluate QTL candidate genes for this trait (Chen et al., 2016). In the present study, the transcriptome data obtained for the 20 BILs and parental cultivars were successfully used to detect QTL candidate genes, without generating substitution or extreme-trait lines.

Similar to previous studies, our data demonstrates that transcriptome analysis is a powerful method for determining the expression profiles reflecting the morphological and physiological traits of an organism. As these expression profiles can be affected by many environmental and genetic factors, and involve complex interactions and signaling pathways, the statistical approaches used to identify functional genes commonly need huge sample sizes to decrease noise. However, using a relatively small number of samples, 20 BILs and two parental cultivars, we successfully identified genes that affect shoot weight, suggesting that a relatively small sample size can still be effective in this type of analysis. However, the need for a large sample size might have been surpassed by the controlled conditions in which rice cultivars were grown. Further, we also used BILs that have similar genetic backgrounds, thus limiting genetic complexity. It is, therefore, important to note that using transcriptomic data from strains with various genetic backgrounds, under variable environmental conditions, might require a larger sample size than that used here to identify functional genes.

## Conclusion

In conclusion, we utilized both QTL and RNA-Seq analyses to identify candidate genes for shoot weight in rice. This analysis revealed that *qLTG3-1* and *SG1* might influence seedling growth rate. Because the environmental conditions and genetic background in our experiments were constant, a large sample size was not required. As this is the first time this unbiased, statistical approach has been used to select candidate genes whose expression is related to shoot weight/seedling growth in rice, additional work is required to better understand the function and regulation of these genes. Because seedling growth is an important trait during direct seeding, this study provides essential information for rice agronomics.

## Author Contributions

AF, TH, NA, SK, MY, CO, and AN conceived and designed the research. TK developed the rice BILs. AF and TH performed the physiological experiments. AN calculated the regression model and selection frequency. AF wrote the manuscript. All authors read and approved the final manuscript.

## Conflict of Interest Statement

The authors declare that the research was conducted in the absence of any commercial or financial relationships that could be construed as a potential conflict of interest.

## Footnotes

1 http://archive.gramene.org/markers/microsat/supp-table18.xls

2 http://statgen.ncsu.edu/qtlcart/WQTLCart.htm

3 ftp://ftp.ensemblgenomes.org/pub/release-24/plants/fasta/oryza_sativa/dna/

4 ftp://ftp.ensemblgenomes.org/pub/release-24/plants/gtf/oryza_sativa/

5 https://www.R-project.org/

6 http://rapdb.dna.affrc.go.jp/

7 http://plants.ensembl.org/index.html

## Abbreviations

BILs: backcrossed inbred lines
eQTLs: expression quantitative trait loci
FPKM: fragments per kilobase per million
ORF: open reading frame
QTLs: quantitative trait loci
RNA-Seq: RNA sequencing
SG1: short grain 1
