# Supplementary material for: Selection of Transcripts Affecting Initial Growth Rate of Rice Backcrossed Inbred Lines Using RNA Sequencing Data
Source: Front Plant Sci. 2018 Dec 20;9:1880. doi: 10.3389/fpls.2018.01880 (PMC6315124; doi:10.3389/fpls.2018.01880)
Supplement: Supplementary file 1 [file Image_1.pdf]

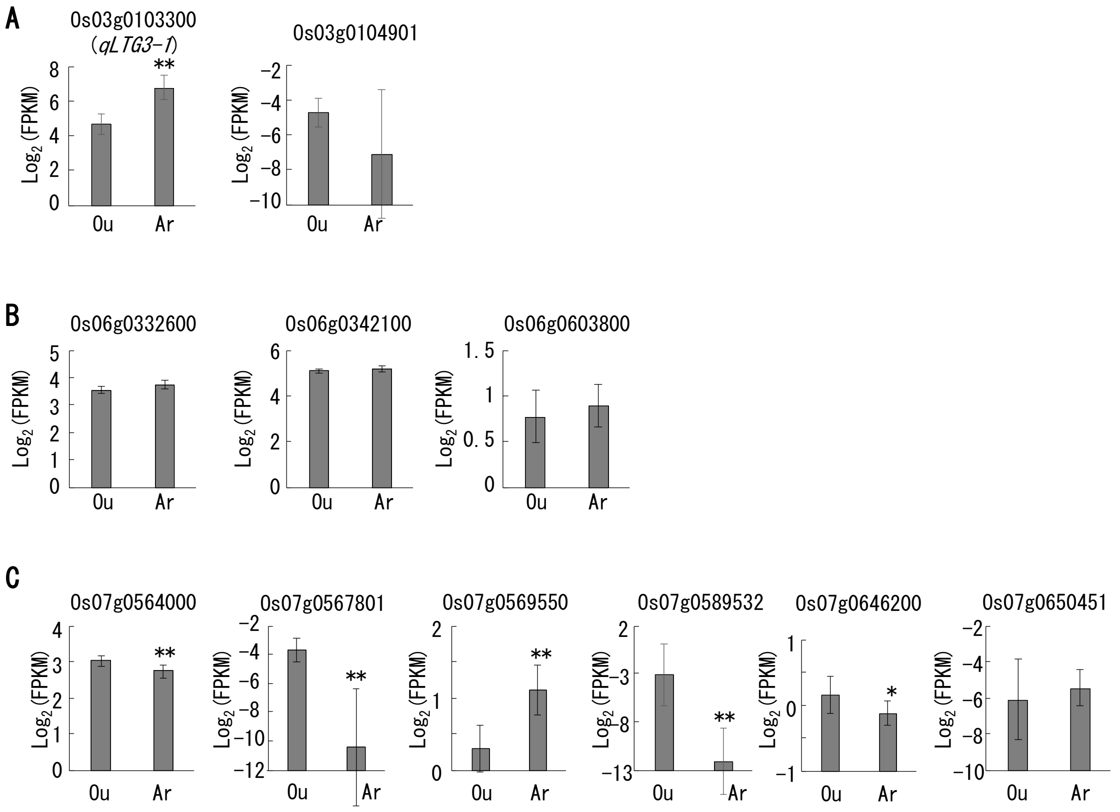

Supplementary Figure 1. Expression of high-frequency genes located at the QTLs for shoot dry weight. The Ouu 365 homozygous (Ou) and Arroz da Terra homozygous (Ar) genotypes in the QTLs are indicated. The genotypes for the genes located at (A) *qSW-3* (Ou: n = 14, Ar: n = 8), (B) *qSW-6* (Ou: n = 18, Ar: n = 4), and (C) *qSW-7* (Ou: n = 12, Ar: n = 9) were classified with the markers qLTG3-1, RM6836, and RM5847, respectively. Data are presented as means ± standard deviation. *$P < 0.05$, **$P < 0.01$.
